# Mesenteric Panniculitis with Raised Alanine Transaminase Levels: A Rare Case Report from Pakistan

**DOI:** 10.7759/cureus.6411

**Published:** 2019-12-18

**Authors:** Samar Mahmood, Asim Sharif, Khushboo Nusrat, Shoukat Ali Samjo, Zaigham Abbas

**Affiliations:** 1 Internal Medicine, Dow University of Health Sciences, Karachi, PAK; 2 Gastroenterology, Ziauddin University Hospital, Karachi, PAK; 3 Gastroenterology and Hepatology, Ziauddin University Hospital, Karachi, PAK

**Keywords:** mesenteric panniculitis, alanine transaminase, alanine aminotransferase, mesenteric thickening, halo sign, pakistan, gastroenterology, mesenteric stranding

## Abstract

Mesenteric panniculitis (MP) is a rare condition that encompasses a spectrum of disease processes characterized by degeneration, inflammation, and scarring of the adipose tissue of the mesentery. The etiology of MP remains unknown; although various causes have been suggested and it has been seen to occur independently as well as in association with other disorders. The clinical manifestations of MP vary over a spectrum, and most patients actually experience no discomfort at all. When present, these clinical presentations vary according to the stage of the disease and may include general symptoms like abdominal pain and weight loss or more specific ones such as an abdominal mass, peritoneal irritation, and ascites. CT findings have emerged to be the gold standard in diagnosis, wherein MP is characterized by localized mesenteric thickening and stranding covering the blood vessels with a characteristic ‘halo sign’, in which the fat around the lymph nodes and blood vessels is spared. Here, we present the case of a 45-year-old male patient who reported to a private hospital in Karachi, Pakistan with non-specific complaints of abdominal pain and vomiting and typical CT and histopathology findings of MP on investigation, as well as abnormally raised alanine transaminase (ALT) levels.

## Introduction

Mesenteric panniculitis (MP), also known as mesenteric lipodystrophy, sclerosing mesenteritis, mesenteric Weber-Christian disease, retractile mesenteritis, and mesenteric fibrosis encompasses a spectrum of disease processes characterized by degeneration, inflammation, and scarring of the adipose tissue of the mesentery [1,2]. Patients with MP have varying clinical manifestations and most experience no discomfort at all. Clinical presentations, when present, vary according to the stage of the disease and may include abdominal pain, weight loss, nausea, and vomiting or, more specifically, an abdominal mass, peritonitis, peritoneal irritation, and ascites [1,3]. The etiology of MP remains unknown; it may occur independently or in association with other disorders. Various causes have been suggested, including autoimmune disorders, infection, trauma (including recent surgery), and ischemia of the mesentery. The condition has a poorly understood link with underlying malignancy as well, which implies its potential paraneoplastic nature, at least in some of the patients [3].

Although very easy to misdiagnose, the diagnosis of MP is increasingly being made correctly thanks to the growing use of abdominal diagnostic imaging [2]. CT findings help the diagnosis, wherein MP is characterized by localized mesenteric thickening and stranding covering the blood vessels with a characteristic ‘halo sign,’ in which the fat around the lymph nodes and blood vessels is spared [4]. The histopathological findings supplement those of the CT scan, broadly comprising of fibrosis, chronic inflammation, and fat necrosis, to variable degrees [5]. The radiological prevalence of the rare condition of MP is reported at 0.16-7.80% with a predisposition for middle and late adulthood (most common in the 50-60 years age group) and a male-to-female ratio of 1.5-1.8:1.0 [3,5]. Although few data are available on the clinical outcomes and response to therapy of patients with MP, the natural course is often benign and self-limiting [5].

In this report, we present the case of a 45-year-old male patient who reported to a private hospital in Karachi, Pakistan with non-specific complaints of abdominal pain and vomiting as well as abnormally raised alanine transaminase (ALT) levels, as discovered on the investigation. With this reporting of the course of his investigation, his characteristic CT, and histopathology findings of MP and the prescribed treatment, we hope to provide additional insight into this limitedly reported condition and encourage its consideration in the workup of seemingly vague gastrointestinal complaints in future.

## Case presentation

A 45-year-old male banker presented at the outpatient department with complaints of intermittent abdominal pain for four weeks; he also mentioned two to three episodes of vomiting that occurred only on the first day of illness. Upon further inquiry, the patient reported as being in a state of good health before this current presentation, when he spontaneously developed diffuse, spasmodic pain of moderate-to-severe intensity in his upper and middle abdomen that was not associated with food intake or any other aggravating factors. The pain had been relieved by painkillers. Although the patient complained of an associated fever, this was not documented. He reported just two to three episodes of vomiting, with no significant historical features. There was no history of diarrhea, bleeding per rectum, burning micturition or cough, or weight loss. His appetite and bowel habits were normal. His past medical, surgical, family, and personal histories were unremarkable.

Upon examination, he was a young healthy looking male of average height and built, vitally stable and afebrile, without any obvious distress. He was alert, oriented to time, place, and person and co-operative. The general physical, respiratory, cardiovascular, musculoskeletal, and central nervous system examinations were unremarkable. The abdominal examination yielded soft, mild central abdomen tenderness and audible gut sounds with no visceromegaly.

**Figure 1:**
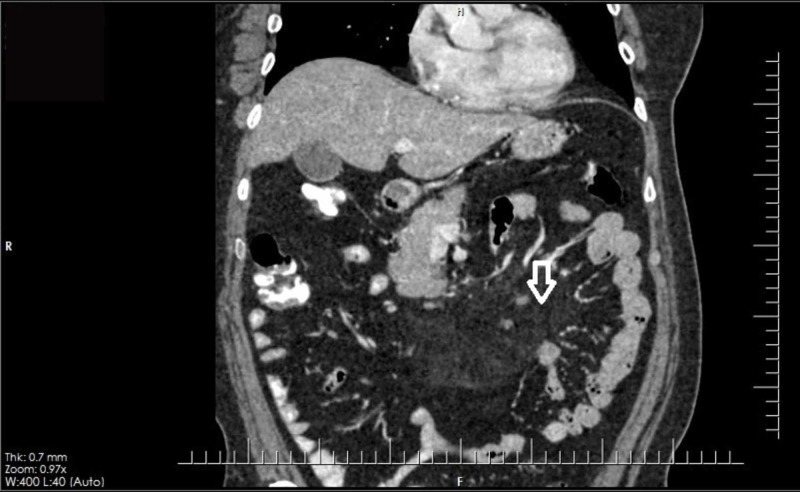
CT coronal view showing ill-defined, mesenteric fat stranding in the center of the abdomen – in the umbilical and supraumbilical locations and encasing the vessels

**Figure 2:**
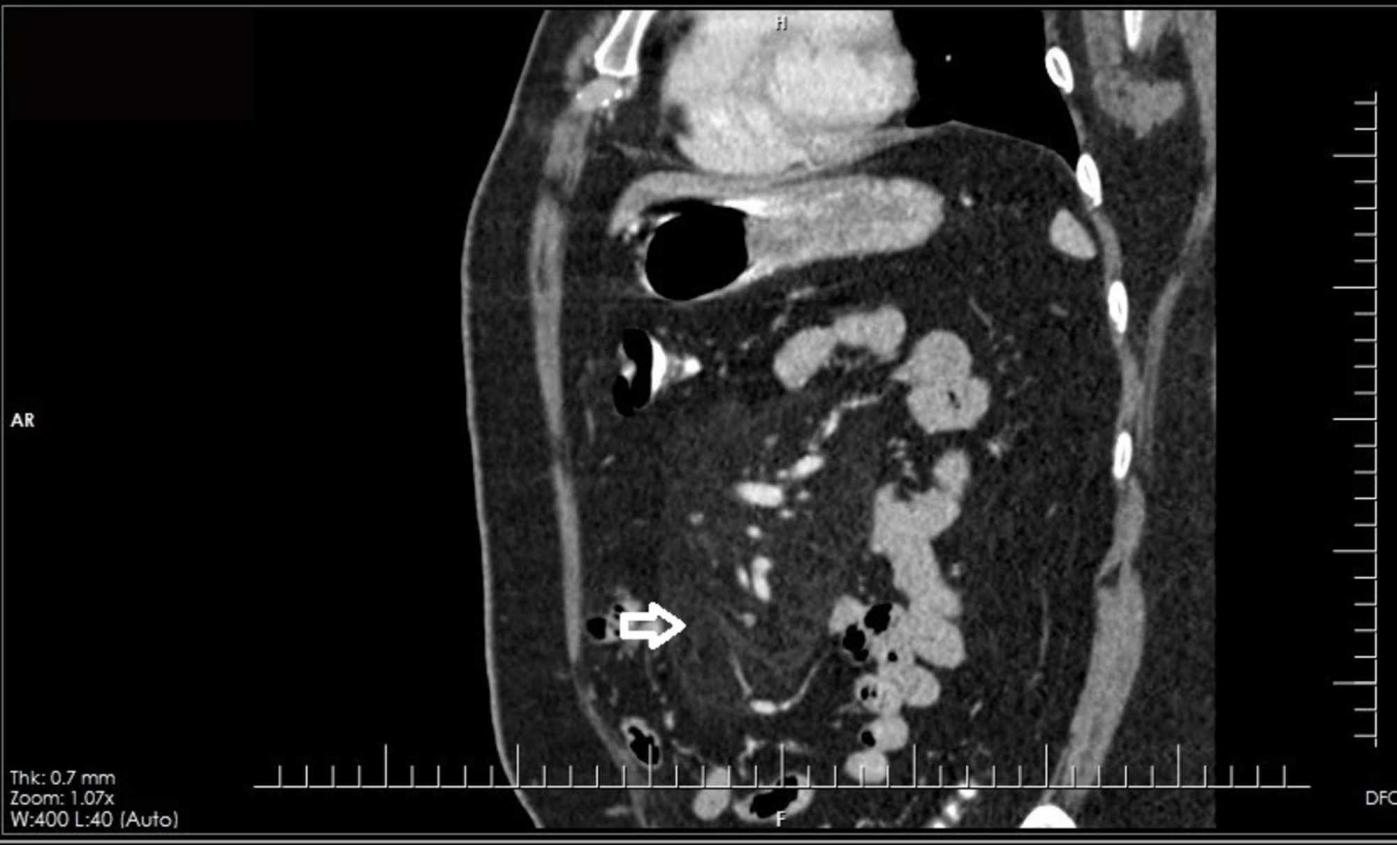
CT sagittal view showing ill-defined, mesenteric fat stranding in the center of the abdomen – in the umbilical and supraumbilical locations and encasing the vessels

His routine blood workup including complete blood counts, urea, creatinine, electrolytes, and urine detailed report and blood culture were all normal. However, his liver function tests yielded abnormally high ALT levels, at 74 IU/L. Viral markers were negative. Creatinine phosphokinase levels, the fasting lipid profile, and pancreatic enzymes were also normal. An abdominal ultrasound revealed no sonographic hepatobiliary pathology. As a part of the evaluation for raised ALT levels, autoimmune markers including IgG4 and ceruloplasmin were negative. Ferritin was around 360 ng/ml, but the rest of the iron profile including transferrin saturation was normal. In view of the patient’s chronic, vague abdominal pain, a CT scan was ordered. The CT scan revealed ill-defined, mesenteric fat stranding in the center of the abdomen, in the umbilical and supraumbilical locations and encasing the vessels, but without vascular narrowing and with few surrounding enhancing lymph nodes, suggestive of mesenteric panniculitis; the rest of the abdomen appeared normal (Figures 1 and 2). To further corroborate the diagnosis, biopsy samples were taken from the omentum as well as the liver laparoscopically, owing to the raised ALT levels. His histopathology report yielded patchy, mild, acute and chronic, non-specific inflammation of the fibroadipose tissue, consistent with the radiological diagnosis; the liver showed no significant inflammation or steatosis. The modified hepatic activity index was 0/18, but there was a fibrous expansion of few portal areas, staged as 1/6 fibrosis. The report showed no granuloma or malignancy.

He was given a two weeks’ course of steroid therapy followed by colchicine 1.2 mg in divided doses for a period of 6 months. He showed a good response within 3-4 weeks and remained symptom-free with no relapse reported so far. Moreover, his ALT levels have consistently remained normal. 

## Discussion

MP, predominantly a disease of the small-bowel mesentery in 90% of the cases, is known to less commonly involve the sigmoid mesentery and, rarely, the mesocolon, peripancreatic region, omentum, retroperitoneum, and pelvis as well [6]. With a mean clinical progression of 6 months (range: six weeks to 12 years), the disease is asymptomatic in 30-50% of the cases [7]. Almost 20% of patients have a more symptomatic debilitating disease, wherein the most common symptom is abdominal pain (68-78%), as experienced by our patient. This may be accompanied by various non-specific symptoms such as anorexia, abdominal fullness (9-26%), nausea and vomiting (6-32%), pyrexia (6-26%), and constipation (6-15%), occasionally single or multiple palpable masses and, exceptionally, rectal bleeding, jaundice, gastric outlet obstruction, and even acute abdomen [6-9]. Such a wide variety of non-specific manifestations means that the treating physicians and surgeons must always carry out a detailed assessment of any patient’s complaints within this spectrum and consider it as a differential in their regular practice.

Although the precise etiology of MP remains unknown, the disease is related to numerous factors such as abdominal trauma or surgery, mesenteric thrombosis, mesenteric arteriopathy, drugs, thermal or chemical injuries, vasculitis, avitaminosis, autoimmune disease, retained suture material, pancreatitis, bile or urine leakage, hypersensitivity reactions, bacterial infections, gallstones, coronary disease, cirrhosis, abdominal aortic aneurysm, peptic ulcers, and even tobacco consumption [8]. The histological course of the disease is much better understood as compared to its etiology and is broadly classified into three stages: mesenteric lipodystrophy, mesenteric panniculitis, and retractile mesenteritis. When into the second stage, histology reveals an infiltrate made up of plasma cells and a few polymorphonuclear leukocytes, foreign-body giant cells, and foamy macrophages. This is when the disease turns symptomatic and the most common symptoms include fever, abdominal pain, and malaise. The final stage shows collagen deposition, fibrosis, and inflammation; the former leads to scarring and retraction of the mesentery, which in turn leads to the formation of abdominal masses and obstructive symptoms [10].

Patients of MP are known to have strong family histories of other autoimmune diseases such as gout, rheumatoid arthritis, and systemic lupus erythematosus as well as various cancers [11]. Moreover, patients of MP themselves usually have significantly higher incidences of concomitant malignancies such as lymphoma, lung cancer, melanoma, colon cancer, renal cell cancer, myeloma, gastric carcinoma, chronic lymphocytic leukemia, Hodgkin’s disease, large cell lymphoma (giant-cell carcinoma), carcinoid tumor, thoracic mesothelioma and breast, ovarian, and endometrial carcinomas [5,6]. Daskalogiannaki et al. found 69.3% of their MP patients as having an associated malignancy, most of which were urogenital or gastrointestinal adenocarcinomas or lymphomas [5]. In addition, they reported the chance of future cancer development during a set 5-year follow-up period to be significantly higher in patients with MP than in the control group [2]. This association between MP and malignancies is poorly understood. Although Kipfer et al. suggested that MP is a non-specific response to underlying abdominal malignancy, this is not unanimously agreed upon and is a theme that needs further exploration [12]. Another reported theory is that patients of MP are generally males from the 50-60 years age bracket, which is a group with an increased likelihood of cancer development in general [2].

Regarding the role of investigations in reaching a diagnosis of MP, laboratory parameters are usually normal with occasional increases in ESR, CRP, and/or evidence of anemia, leukocytosis, and hypoalbuminemia [13]. Albeit sparingly reported, it is now believed that liver dysfunction associated with mesenteric lipodystrophy might occur more frequently than previously thought. Since symptoms of mesenteric lipodystrophy are relatively subtle or even absent, they may not be recognized by patients or clinicians and thus the association is not reported often [14]. Although the literature mentions a case of MP with associated acute cholestasis (serum alkaline phosphatase (ALP) level higher than 2.5 times the upper limit of normal (ULN) and ALT/ALP ratio fewer than 2 times the ULN), our case was unusual in its presentation of associated, raised ALT levels [14]. The differential diagnoses devised for MP in the past include an extensive list of conditions, such as infectious diseases like tuberculosis and histoplasmosis, Whipple’s disease, amyloidosis, peritoneal mesothelioma, reaction to adjacent cancer or chronic abscess, retroperitoneal sarcoma, lymphoma, carcinoid, and desmoid tumors [6]. Hence, a wide array of laboratory investigations is generally ordered to rule out many of these considered conditions in each instance of MP, as was done in our case as well.

As for the tool of a definitive diagnosis, CT scanning has emerged to be the gold standard. There are five known cardinal signs of MP on CT: a well-defined mesenteric fatty mass, a mass with higher density than the adjacent abdominal fat, the presence of blood vessels and small lymph nodes inside the mass, a “halo sign” or fat ring sign around the lymph nodes and vessels, and the presence of a hyperattenuating stripe around the mass known as a pseudocapsule [4]. Most studies in the past have concluded the prevalence of at least three of these on a scan as definitive, a criterion met by our case as well. In a study by Al-Omari et al. comparing the morphological differences on CT between malignancy- and non-malignancy-associated MP, it was found that, in paraneoplastic conditions, MP was associated with more hyperdense fat (misty mesentery), a markedly higher frequency of pseudocapsule formation, and a longer mean craniocaudal diameter of the present mass than in cases of non-malignant MP [4].

Various drugs have been used to treat MP, including corticosteroids, colchicine, cyclophosphamide, azathioprine, tamoxifen, emetine, and thalidomide, as well as hormonal therapies and radiotherapy, all with varying degrees of success [15]. In general, treatment is reserved for symptomatic cases, as most cases are known to resolve spontaneously. However, palpable masses may often be found between two and 11 years after diagnosis, especially in patients with associated comorbidity that are left untreated if found incidentally. Surgical resection may be attempted if medical therapy fails or in the presence of life-threatening complications such as bowel obstruction or perforation [6]. Interestingly, a study from last year has displayed the effective role of non-steroidal anti-inflammatory drugs (NSAIDs) and antibiotics in the treatment of idiopathic MP, indicating that the trigger event might be a self-limiting inflammation or infection that spreads from adjacent structures to the mesenteric adipose tissue in most patients. This highlights how the natural history of MP across the rest of the world may be different from that in Western countries, presumably due to lower sanitary conditions, infections with several unidentified microorganisms, and genetic differences. Therefore, we see the need for epidemiological studies from Asian and African countries to be conducted, in order to obtain more accurate epidemiologic data on MP [15].

## Conclusions

We presented a rare case of MP in a 45-year-old man, with nonspecific presenting symptoms of abdominal pain, vomiting, and undocumented fever as well as raised ALT levels. This report discusses the etiology, associations, investigations, and treatment options for MP in detail, highlighting the importance of its consideration as a differential by all physicians and surgeons evaluating patients for similar gastrointestinal complaints or aimed/incidental radiological findings.
